# Rapid Quantification of Major Volatile Metabolites in Fermented Food and Beverages Using Gas Chromatography-Mass Spectrometry

**DOI:** 10.3390/metabo7030037

**Published:** 2017-07-27

**Authors:** Farhana R. Pinu, Silas G. Villas-Boas

**Affiliations:** 1 The New Zealand Institute for Plant & Food Research Limited, Private Bag 92169, Auckland 1142, New Zealand; 2 School of Biological Sciences, University of Auckland, Private Bag 92019, Auckland 1010, New Zealand; s.villas-boas@auckland.ac.nz

**Keywords:** Ethanol, acetic acid, fermented beverages, balsamic vinegar, sourdough, wine

## Abstract

Here we present a method for the accurate quantification of major volatile metabolites found in different food and beverages, including ethanol, acetic acid and other aroma compounds, using gas chromatography coupled to mass spectrometry (GC-MS). The method is combined with a simple sample preparation procedure using sodium chloride and anhydrous ethyl acetate. The GC-MS analysis was accomplished within 4.75 min, and over 80 features were detected, of which 40 were positively identified using an in-house and a commercial mass spectrometry (MS) library. We determined different analytical parameters of these metabolites including the limit of detection (LOD), limit of quantitation (LOQ) and range of quantification. In order to validate the method, we also determined detailed analytical characteristics of five major fermentation end products including ethanol, acetic acid, isoamyl alcohol, ethyl-L-lactate and, acetoin. The method showed very low technical variability for the measurements of these metabolites in different matrices (<3%) with an excellent accuracy (100 ± 5%), recovery (100 ± 10%), reproducibility and repeatability [Coefficient of variation (CV) 1–10%)]. To demonstrate the applicability of the method, we analysed different fermented products including balsamic vinegars, sourdough, distilled (whisky) and non-distilled beverages (wine and beer).

## 1. Introduction

**Figure 1:**
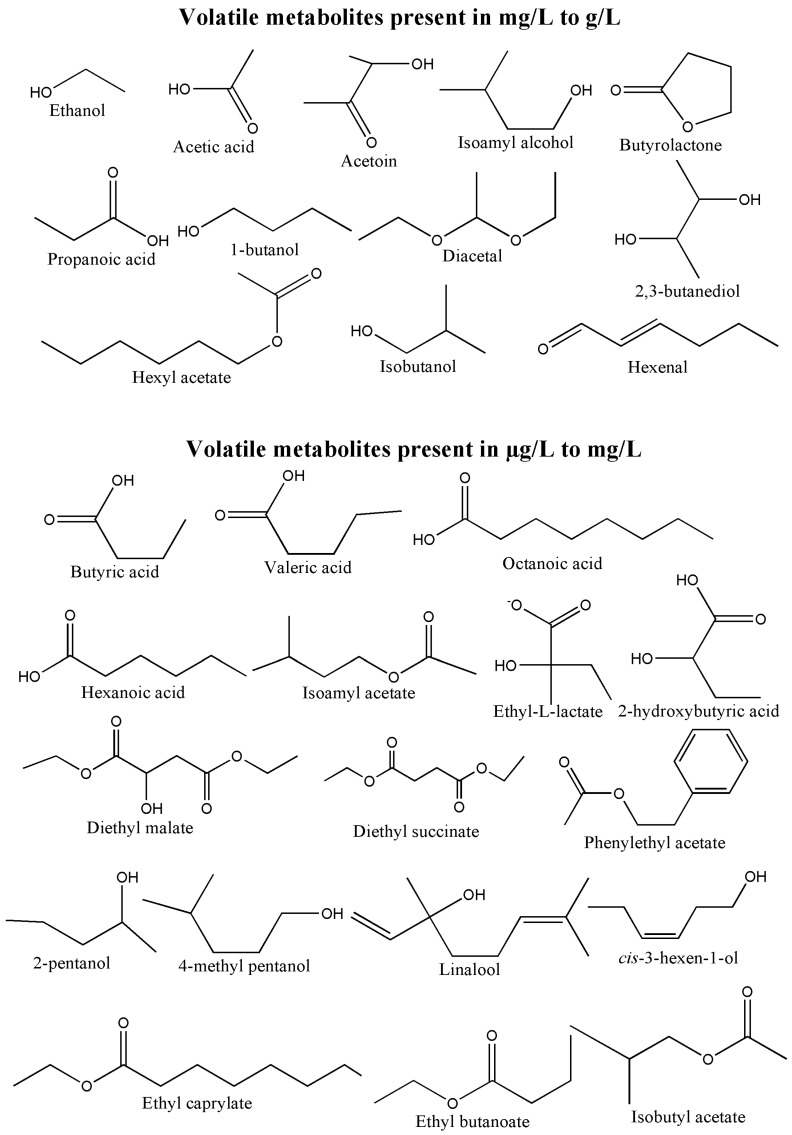
The structures of well-known major volatile metabolites present in different fermented food and beverages.

Gas chromatography and mass spectrometry (GC-MS) in one of the most mature analytical technologies that provides excellent chromatographic resolution due to the high sensitivity and specificity of mass spectrometry [1]. GC-MS has always been the most preferred analytical platform for the analysis of volatile metabolites present in different biological samples including food and beverages. Therefore, many methods are already available that make use of headspace (HS) and solid-phase micro extraction (SPME) techniques in order to quantify volatile compounds accurately in fermented food and beverage samples [2,3,4,5,6]. However, HS analysis requires a large sample volume and SPME requires expensive fibres for effective extraction of volatile metabolites. On the other hand, liquid injection GC-MS offers a cheaper and more straightforward option for the analysis of major fermentation end products (e.g., ethanol, acetic acid, higher alcohols, esters and volatile fatty acids, Figure 1) with an advantage of being able to identify other volatile metabolites easily that are commonly found in fermented products.

Volatile metabolites, usually present in ng/L to g/L, play very important roles in sensory properties of different fermentation products (Figure 1). Although some of them are already present in the raw materials (e.g., fruits, grains), they can also be produced by the activities of different microorganisms including bacteria, yeast and other fungi. Therefore, their concentrations increase significantly in the final fermented product, as compared to the initial raw material. For instance, ethanol and acetic acid are the two major fermentation end products found in various food and beverages. Therefore, quantification of ethanol and acetic acid in food and alcoholic beverages is well established in biological samples using different analytical and enzymatic approaches [7,8,9,10]. But most of these methods do not offer the same sensitivity and, most importantly, accuracy if compared to GC-MS. As such, dichromate oxidation spectrophotometry [11], densimetric analysis [12], refractive index method [12,13] and near-infrared spectroscopy [14,15] have been used for quantification of ethanol for many years. However, these methods require large sample volume and/or pre-treatment (e.g., distillation) of samples that make them not ideal for small sample sizes [16]. Enzymatic assays are also commonly used to quantify ethanol and acetic acid in biological samples [8,9,17]. However, the reproducibility of the enzyme-based methods is not good due to instability of enzymes. Moreover, enzymatic reactions are highly influenced by the matrix of the samples. On the other hand, several chromatographic methods have been reported for quantification of ethanol and acetic acid, mostly using high performance liquid chromatography (HPLC) and gas chromatography coupled to flame ionisation detector (GC-FID) [16,18,19,20,21]. HPLC methods provide poor chromatographic resolution when analysing complex matrices [16], and the commonly used detectors in HPLC-based methods (UV and refractive index) are unable to distinguish co-eluting metabolites. Similarly, FID presents very low specificity [20]. 

Given that the concentration of volatile metabolites varies widely (Figure 1) in different fermented products, it is always a challenging task to develop the analytical pipelines that would allow simultaneous quantification of them (as many as possible) in a single run using comparatively a small amount of sample and minimum sample preparation. 

Although many methods are already available for the analysis of volatile compounds, however, there is still an ongoing demand for developing a method that is quick, inexpensive and requires only a small amount of samples, yet accurate. Therefore, we aimed to develop such a method for the global analysis of volatile metabolites. Here, we present a direct injection GC-MS method for the quantification (and profiling) of different fermentation end products in food products and beverages, which combine the high specificity of electron impact (EI) mass spectrometry with the high reproducibility and high chromatographic resolution of gas chromatography.

## 2. Results and Discussion

### 2.1. Method Validation

**Figure 2:**
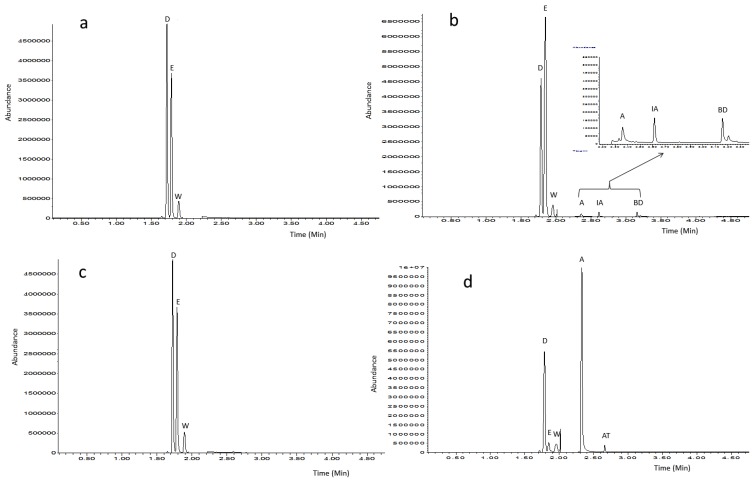
Typical gas-chromatography-mass spectrometry (GC-MS) chromatograms obtained from the analysis of beer (**a**), wine (**b**), whisky (**c**) and vinegar (**d**) ethyl acetate extracts. D = D_4_-methanol, E = ethanol, W = acetonitrile (from wash solvent), A = acetic acid, IA = isoamyl alcohol, BD = 2,3-butanediol and AT = acetoin.

Although our initial aim was to develop a rapid, reproducible and inexpensive GC-MS method to accurately quantify ethanol and acetic acid simultaneously in various fermented food products and beverages using only 100 µL of sample (Figure 2), we were also able to detect over 70 features only within 4.75 min while using 1000 µL of sample (Table 1). Therefore, this method has potential for volatile metabolite profiling when large volume of biological samples are not available.

We also identified over 40 volatile metabolites (Table 1) including 14 alcohols (higher alcohols and one terpene alcohol, linalool), 10 volatile organic acids, 12 esters (both ethyl and acetate esters) and five aldehyde and ketones using both reference standards (in-house MS library) and NIST (National Institute of Standards and Technology) library. Therefore, this method clearly shows its potential for the determination of different groups of volatile compounds. To the very best of our knowledge, this is the first method that covers the quantification of a wide range of volatile compounds only within 5 min using small amount of samples (100–1000 µL). 

We determined different analytical parameters of all 40 metabolites including the limit of detection (LOD), limit of quantitation (LOQ) and range of quantification. We obtained excellent linearity for all the metabolites (Table 1). Moreover, LOD varies from 0.1 mg/L to 20 mg/L, while LOQ was within 0.5–50 mg/L depending on the chemical properties of metabolites. For instance, LOD of ethanol and acetic acid was 0.5 mg/L and 0.4 mg/L respectively, and LOQ was 2 mg/L for ethanol and 1.5 mg/L for acetic acid when only 100 µL samples were used, which make this a more sensitive method for analysis of these common fermentation products than other published GC-FID and HPLC methods [7,10,18,20]. Therefore, this method is indeed suitable for the quantification of different metabolites that are present in mg/L to g/L. It is noteworthy that other published methods make use of at least 10–50 mL of samples using either HS or SPME extraction and sample concentration step in order to quantify volatiles present in µg/L or below [2,5,22]. On the contrary, we only used 100–1000 µL of samples using a very simple sample preparation protocol to achieve these extensive ranges of quantification (0.5–350000 mg/L) of 40 different volatile metabolites. Moreover, the samples were injected to GC at 100:1 split ratio. Therefore, it is possible to achieve even better LOD and LOQ by increasing the volume of samples extracted by ethyl acetate as well as by reducing the split ratio [23].

Our chosen internal standard was D4-methanol, which is an unnatural compound that elutes separately before most of the metabolites, making it an appropriate internal standard for this method (retention time in Table 1). Excellent repeatability and reproducibility results were obtained for five major volatiles (ethanol, acetic acid, ethyl-L-lactate, isoamyl alcohol and acetoin) with less than 4.6% technical variability (RSD) using seven different matrices (Table 2). Similarly, the intra- and inter-day variability for all these metabolites was also below 5% (Table 2). All these results indicate that the precision of our method is high. In addition, the method showed excellence accuracy and the overall recovery of five major fermentation end products in five different matrices was 100.0% ± 6.0% (Table 3), which confirms that this method is able to quantify these volatile metabolites accurately in wide ranges of fermented beverages and food products.

The choice of extraction solvents depends on the polarity of the metabolites of interest and organic solvents are mostly used as they allow the precipitation of proteins present in the samples [24]. While aqueous solutions of methanol, acetonitrile, and isopropanol are suitable for the extractions of polar metabolites [24], pure organic solvents (e.g., ethyl acetate, hexane) are able to extract volatile fraction of metabolome of a given biological samples. We choose ethyl acetate as the solvent for extraction of volatile compounds from water-based samples because it has very low solubility in water (8.3 mg/100 mL) [25] and it has high partition co-efficient for ethanol, acetic acid and other volatiles in aqueous solution [20]. In addition, anhydrous NaCl was added to the sample during extraction in order to increase the polarity of the aqueous layer and maximise extraction of volatile compounds [26]. By comparing samples extracted in presence and absence of NaCl, we observed a much better reproducibility and accuracy of analysis when NaCl was used (data not shown).

### 2.2. Applicability of the Method

We analysed six different types of fermented food and beverages including sourdough (*n* = 3), balsamic vinegars (*n* = 6), whiskies (*n* = 3), beers (*n* = 3), red (*n* = 3) and white wines (*n* = 3) to determine the applicability of the method for the quantification of major volatile metabolites (Table 4). Although this method is able to detect and quantify over 40 volatiles present in a sample, we identified and successfully quantified 18 of the volatile metabolites in the samples analysed in this study. Among them, only five metabolites were present in all the samples including ethanol, acetic acid, acetoin, 2,3-butanediol and phenylethyl alcohol. Their concentrations in various fermented food and beverages (65–33078 mg/L) also indicates that these are the major fermentation end products in those samples. 

Ethanol was undoubtedly the most abundant and common metabolites present in concentrations well above g/L in all the samples analysed, which was expected. For instance, ethanol concentrations in the wines ranged from 93.34 g/L to 108.25 g/L, which were very similar to the concentrations mentioned in the wine labels (recovery: 98.89–101.61%) (Table 4). Similarly, we also determined the ethanol content in three distilled spirits (307.32–372.98 g/L) and three different beers (36.64–55.78 g/L) (Table 4). These values were very close to the concentrations provided by the manufacturers, which again shows the potentiality of using this method for the analysis of ethanol in many alcoholic beverages with wide range of ethanol concentrations. In addition, we also determined the ethanol concentration in six balsamic vinegars (1.24–2.27 g/L) and three sourdough samples (1.5–1.75 g/L) (Table 4), which indicates that this method also can be used to quantify ethanol in food products with comparatively low ethanol content. 

Acetic acid is the second most common and abundant volatile metabolite present in different fermented food and beverages. Table 4 shows the concentration of acetic acid in alcoholic beverages, sourdough and vinegar samples. The acidity of balsamic vinegars was mentioned as approximately 6% _v/v_ in the bottles and our data show that acetic acid concentration varied from 5.24% _v/v_ (55.02 g/L) to 6.31% _v/v_ (66.25 g/L), which were very close to the value mentioned by the manufacturers. The acetic acid concentration in beer and whisky samples ranged from 0.11 g/L to 0.37 g/L, and it was mostly below 0.57 g/L for both red and white wines, which is expected because acetic acid is considered an off-flavour in many beverages (Table 4). However, one red wine showed more than 0.88 g/L of acetic acid, which might produce a pungent flavour in the wines [27].

The overall volatile metabolite profile largely depended on the type of samples analysed during this study. For instance, the concentrations of alcohol and higher alcohols were comparatively higher in whisky samples as expected, while almost no volatile organic acids and esters (except diethyl succinate) were detected in them. It is well known that whisky is a distilled beverage, thus only the major alcohols remained in them after the distillation process [28]. Although some volatile compounds usually develop during maturation [28], it is possible that most of the organic acids and esters are present in too low concentrations to be detected by our method. In addition to this, wines also had the most distinct volatile metabolite profiles and some of the metabolites were uniquely present in different types of wines. For example, two c6 compounds (cis-3-hexen-1-ol and hexyl acetate), also known as green leaf volatiles, were only detected in wine samples. However, they could only be quantified in white wines (Table 4). All these white wines were produced in New Zealand where machine harvesting is mostly used method. Therefore, these volatile compounds most probably originated during the crushing process of the grapes along with the leaves [29] and a minor amount also can be produced by the metabolic activities of wine yeasts [30]. Similarly, a naturally occurring terpene alcohol, linalool, which contributes to the floral notes in wines [31], was also only detected in both of types of wines, although under limit of quantification (Table 4).

## 3. Materials and Methods 

### 3.1. Chemicals

All chemicals used in this study were of analytical grade. Internal standard tetradeuteromethanol (D4-methanol; 99.8%) was purchased from Cambridge Isotope Laboratories, Inc. (Andover, MA, USA). The solvent anhydrous ethyl acetate (99.8%), ethanol standard (99.5%), and all other volatile metabolite standards were obtained from Sigma-Aldrich (St. Louis, MO, USA), except acetone (99%) and acetic acid (99.8%), which were purchased from Biolab (Scoresby, Australia). All solutions were prepared using Grade 1 water (BARNSTEAD^®^ NANOpure Diamond^TM^ Water Purification System, Waltham, MA, USA) or absolute ethanol or absolute methanol (UNIVAR, AJAX FINECHEM, Auckland, New Zealand). 

### 3.2. Sample Preparation

We used only 100 µL of sample to quantify ethanol and acetic acid in different samples. However, 1000 µL of samples were needed to determine the concentrations of other volatile metabolites. The samples were mixed with 10 µL of internal standard (D4-methanol). Approximately 50–300 mg of anhydrous sodium chloride salt was added to the mixture until saturation followed by intense mixing using a vortex mixer for 10–15 s. Then, 500 µL of ethyl acetate was added to the mixture and mixed vigorously for 1 min using a vortex mixer. The samples were then centrifuged for 2 min at 2000× *g* to separate the aqueous and non-aqueous layers. Approximately 200 µL of the upper non-aqueous phase was transferred to a 2–mL GC-MS vial for analysis.

### 3.3. GC-MS Analysis

The ethyl acetate extracts were analysed using an Agilent GC 7890 coupled to a MS 5975 (Agilent Technologies, CA, USA) with a quadrupole mass selective detector (electron impact ionisation; positive mode) operated at 70 eV. A Zebron ZB-1701 (Phenomenex), 30 m × 250 µm (internal diameter) × 0.15 µm (film thickness), with 5 m guard column was used for the analysis. 1 μL of sample was injected into the GC under split mode at a 100:1 split ratio under constant flow of 48.851 mL/min on column. Electron impact ionisation (EI) The temperature of the inlet was kept at 180 °C. The GC oven temperature was initially held at 50 °C for 1 min, and then raised to 200 °C at 40 °C/min. The total running time for this method was 4.75 min. The equilibration time was set to 2 min. The interface and quadrupole temperatures were 230 °C and 150 °C respectively. The MS detector was turned off between 2.03 min to 2.21 min to offload ethyl acetate peak. The MS was operated in scan mode with a mass range of 30 to 250 a.m.u. The reference ion (*m/z*) used for identification and quantification of target compounds as well as internal standard are shown in Table 1. Moreover, we also created an in-house MS library using the standard metabolites and this library contains information about retention time and mass spectra information of over 40 different volatile metabolites commonly found in fermented food and beverages detectable by this method. 

### 3.4. Validation of the Method

The validation of the method was carried out using standard solutions of five major fermentation end products including ethanol, acetic acid, ethyl-L-lactate, isoamyl alcohol and acetoin. Both reproducibility and repeatability of the method were determined using standard solutions of the five major volatile compounds, beer, synthetic wine (mimicking a real wine with 94.68 g/L ethanol, 1 g/L glucose, 1 g/L fructose, 4 g/L malic acid, 2 g/L citric acid, 1 g/L succinic acid, 0.5 g/L lactic acid), red wine, white wine, whisky and balsamic vinegar samples. To assess the repeatability, the same sample was injected five times into the GC-MS in sequence. The reproducibility was assessed by extracting five replicates of each sample with ethyl acetate as described above and injected in sequence into the GC-MS. Intra- and inter-day variability was also assessed using five standard solutions. For the determination of intra-day variability, two replicates containing extracted volatiles were injected three times within a day and one sample was kept at room temperature, while the other sample was stored at −20 °C for three consecutive days. These samples were injected into the GC-MS every day three times in order to determine the inter-day variability. The recovery of five volatile compounds was obtained using synthetic wine, spiked samples (red and white wines), and standard solutions in water.

In addition, limit of detection (LOD) and limit of quantitation (LOQ) of 40 metabolites were also determined and a signal-to-noise ratio of 3:1 and 10:1 were considered as LOD and LOQ respectively [32]. 

### 3.5. Application of the Method

Quantification of the major volatile metabolites was performed by creating calibration curves within the ranges of concentrations present in the fermented food and beverages. We then analysed commercial (obtained for local supermarkets) beers (*n* = 3), red wines (*n* = 3), white wines (*n* = 3), whisky (*n* = 3), balsamic vinegars (*n* = 3) and sourdough (*n* = 3) in triplicate to demonstrate the applicability of the method for the quantification of fermentation end products in wide ranges of fermented beverages (Supplementary Table 1). A small amount (~ 10 mg) of activated charcoal was added to the ethyl extracts of red wines, dark beers and balsamic vinegars to overcome matrix effect due to the presence of high concentrations of anthocyanin and polyphenolic compounds. A sample dilution (10×) of distilled spirits was performed while quantifying ethanol in order to fit their concentrations within the linearity range. 

## 4. Conclusions

Here we report the first liquid injection GC-MS method for the quantification of major volatile metabolites present in fermented food and beverages. We obtained excellent accuracy, linearity and reproducibility for quantification of different compounds in sourdough, beers, wines, whisky and balsamic vinegar samples. This method is inexpensive, simple and robust, which makes it suitable for rapid quantitation of many volatiles in different types of fermented foods and beverages. We acknowledge the fact that many other methods for aroma compound analysis are available and previously published, but none of the methods are suitable when small amount of samples are available from an experiment. Moreover, all those published methods requires longer time (>20 min and often over 60 min) to complete the GC-MS analysis. In comparison, the method described here only requires 100–1000 µL of samples and analysis is accomplished below 5 min, which make this proposed method unique and suitable for rapid analysis of volatiles in different types of biological samples. There is an ongoing demand of such method within scientific community. Currently, this method is able to quantify only volatiles present in mg/L to g/L, however, the sample preparation step can be further optimised in order cover the µg/L ranges of concentration. Overall, this method is also suitable for both targeted and untargeted volatile profiling approach.

## Figures and Tables

**Table 1:** The analytical characteristics of volatile metabolites detected and identified by Gas-Chromatography and Mass-spectrometry method.

Compounds	Retention Time (min)	Reference Ion	Detection Limit (mg/L)	Quantification Limit (mg/L)	Range of Quantification (mg/L)	Regression Line (*n* = 5)	Coefficient (*r*^2^)
Alcohols							
D4-methanol (Internal standard)	1.780	33	-	-	-	-	-
Methanol	1.786	32	5	10	100–80000	y = 17.926x − 1.528	0.9999
Ethanol^*^	1.847	31	0.5^*^ <0.1^+^	2^*^ <1^+^	1–350000	y = 4.321x − 1.237^*^ y = 2.129x − 0.987^+^	0.9999^*^ 0.9970^+^
Isobutanol	2.280	43	5	10	10–50000	y = 4.219x − 0.8876	0.9987
1-butanol	2.417	56	5	10	10–20000	y =6.2622x − 0.9914	0.9979
Isoamyl alcohol	2.698	55	5	10	10–80000	y = 9.9944x − 1.41	0.9950
4-methyl pentanol	2.729	69	4	10	10–50000	y = 10.957x − 1.1913	0.9975
*cis*-3-hexen-1-ol	3.154	41	12	40	40–6000	y = 4.321x − 0.875	0.9931
2,3-butanediol	3.204	57	8	20	20–10000	y = 5.218x − 1.102	0.9901
*trans*-3-hexen-1-ol	3.200	67	20	50	50–7000	y = 2.135x − 0.2981	0.9912
2-pentanol	3.497	45	8	12	12–50000	y = 20.032x − 0.2981	0.9996
1,3-propandiol	3.569	57	10.6	25	25–10000	y = 0.0164x + 0.1013	0.9968
1-phenylethyl alcohol	4.237	107	10	20	20–20000	y = 0.2937x + 7.2903	0.9976
2-phenylethyl alcohol	4.508	91	10	20	20–20000	y = 0.392x + 8.9852	0.9930
Aldehydes and ketones							
Acetone	1.962	58	3	8	8–50000	y = 0.1551x + 0.8478	0.9945
Acetoin	2.721	43	1	6	6–20000	y = 0.1352x + 0.5321	0.9951
Hexenal	2.804	56	3	8	8–10000	y = 1.098x + 0.0251	0.9904
2-hexenal	3.175	43	1	3	3–9000	y = 2.198x + 0.984	0.9913
Butyrolactone	3.963	42	ND	ND	ND	ND	ND
Volatile acids							
Acetic acid^*^	2.346	43	0.4^*^ <0.1^+^	1.5^*^ <0.5^+^	1.5–50000	y = 0.1247x + 0.875^*^ y = 0.0987x + 0.654^+^	0.9956^*^ 0.9942^+^
Propanoic acid	2.890	74	1	5	5–10000	y = 0.0756x + 0.5821	0.9986
Isobutyric acid	3.053	73	1	2.5	2.5–12000	y = 0.1429x + 2.3651	0.9972
Butyric acid	3.249	60	1	2	2–10000	y = 0.1049x + 4.2899	0.9980
Isovaleric acid	3.426	60	1	5	5–8000	y = 0.1256x + 1.2098	0.9976
Valeric acid	3.674	60	0.9	2	2–9500	y = 0.1109x + 5.1481	0.9993
Hexanoic acid	4.056	60	4	8	8–9500	y = 0.1142x + 2.6098	0.9991
3-hydroxybutyric acid	4.281	60	5	10	10–10000	y = 0.0253x + 0.0138	0.9990
Heptanoic acid	4.580	73	9	16	16–30000	y = 0.345x + 4.219	0.9870
Octanoic acid	4.690	60	8	14	14–20000	y = 0.536x + 5.453	0.9840
Esters							
Ethyl isobutyrate	2.508	43	8	12	12–10000	y = 0.536x + 5.453	0.9912
Isobutyl acetate	2.600	43	8	12	12–10000	y = 0.536x + 5.453	0.9943
Ethyl butanoate	2.700	71	8	12	12–10000	y = 0.536x + 5.453	0.9950
Pyruvic aldehyde dimethyl acetate	2.901	75	8	12	12–10000	y = 0.536x + 5.453	0.9933
Ethyl-L-lactate	3.030	75	1	5	5–20000	y = 0.0545x + 0.0953	0.9985
Isoamyl acetate	3.102	70	2	4	4–20000	y = 0.0786x + 0.128	0.9956
Ethyl caproate	3.620	88	8	15	15–10000	y = 0.0037x + 0.0058	0.9993
Hexyl acetate	3.706	84	1	5	5–15000	y = 1.235x − 0.986	0.9912
Ethyl caprylate	4.399	88	4	6	6–10000	y = 0.3509x + 3.0032	0.9943
Diethyl succinate	4.580	101	1	5	5–10500	y = 0.4808x + 2.8397	0.9958
Diethyl malate	5.002	117	1	5	5–10000	y = 0.5632x + 1.298	0.9987
Others							
Linalool	4.123	71	5	10	10-5000	y = 4.437x − 1.235	0.9965

* using 100 µL of sample; ^+^ using 500 µL of sample and ND = not determined.

**Table 2:** The precision of quantification of major fermentation end products using gas chromatography-mass spectrometry (GC-MS) analysis in different matrices.

Matrix	Ethanol		Acetic Acid		Ethyl-L-lactate		Isoamyl Alcohol		Acetoin	
	Precision (RSD %)		Precision (RSD %)		Precision (RSD %)		Precision (RSD %)		Precision (RSD %)	
	RT	RP	RT	RP	RT	RP	RT	RP	RT	RP
Standard solution (Intra-day)	0.8	1.1	0.8	1.0	1.2	1.6	0.7	1.1	1.3	1.7
Standard solution (Inter-day)	3.4	4.2	3.8	4.4	2.0	2.5	1.8	2.1	2.5	4.5
Beer	2.7	2.5	1.3	2.5	2.0	3.2	1.5	2.1	3.2	3.6
Red wine	1.8	2.3	2.2	2.6	3.0	3.2	2.4	4.1	2.3	3.5
Synthetic wine	1.1	1.6	2.0	2.6	3.1	3.3	2.1	2.6	3.3	3.9
White wine	1.5	2.1	2.5	3.0	2.8	3.6	3.2	4.5	3.5	4.6
Whisky	1.7	1.8	3.4	4.1	ND	ND	2.1	2.7	3.8	4.2
Vinegar	1.2	1.3	1.3	1.6	3.4	3.9	4.1	4.5	2.2	3.5

RSD = Relative standard deviation; RT = Repeatability (*n* = 5); RP = Reproducibility (*n* = 5) and ND = not detected.

**Table 3:** The recovery of major volatiles in five different matrices.

Matrix						
		Standard solution 1	Standard solution 2	Spiked synthetic wine	Spiked red wine	Spiked white wine
Ethanol	Actual concentration (mg/L)	7.89	394.5	94680.0	149910.0	126260.0
	Determined concentration (mg/L)	7.93 ± 0.22	394.3 ± 0.76	94780.2 ± 340.3	151410.2 ± 1200.0	126220.3 ± 980.3
	**Recovery (%)**	**101.51**	**99.95**	**100.10**	**101**	**99.97**
Acetic acid	Actual concentration (mg/L)	5.05	630 .0	450.0	880	1200.0
	Determined concentration (mg/L)	5.07 ± 0.03	629.2 ± 9.7	447.5 ± 5.8	885.9 ± 9.5	1208.2 ± 7.4
	**Recovery (%)**	**100.39**	**99.87**	**99.44**	**100.67**	**100.67**
Ethyl-L-lactate	Actual concentration (mg/L)	10.50	30.45	70.25	500.60	1000.2
	Determined concentration (mg/L)	10.95 ± 0.13	30.50 ± 0.25	69.88 ± 0.79	515.55 ± 20.2	1062.8 ± 11.1
	**Recovery (%)**	**104.28**	**100.16**	**99.47**	**102.98**	**106.25**
Isoamyl alcohol	Actual concentration (mg/L)	20.5	500.0	1500.0	5250.0	2500.5
	Determined concentration (mg/L)	20.9 ± 3.5	501.8 ± 10.3	1450.3 ± 30.5	5215.8 ± 12.9	2543.5 ± 50.6
	**Recovery (%)**	**101.95**	**100.36**	**96.67**	**99.34**	**101.72**
Acetoin	Actual concentration (mg/L)	12.5	50.0	250.5	1045.0	555.0
	Determined concentration (mg/L)	12.6 ± 1.5	49.3 ± 0.55	251.5 ± 6.5	1032.1 ± 22.1	561.8 ± 8.1
	**Recovery (%)**	**100.8**	**98.52**	**100.40**	**98.76**	**101.22**

**Recovery (**%**)** is shown in bold.

**Table 4:** Concentration of major volatile metabolites present in different fermented food and beverage.

Metabolite	Concentration In Fermented Food and Beverages (mg/L)					
	Sourdough (*n* = 3)	Balsamic vinegars (*n* = 6)	Beer (*n* = 3)	Red wine (*n* = 3)	White wine (*n* = 3)	Whisky (*n* = 3)
Ethanol	1750.85 ± 200.18	1900.78 ± 750.47	48730.98 ± 6000.99	107150.25 ± 5500.51	95890.45 ± 8700.64	330780.89 ± 90000.64
Acetic acid	1398.55 ± 65.99	5910.62 ± 300.78	300.12 ± 20.66	455.17 ± 145.10	260.18 ± 79.24	50.29 ± 32.99
Acetoin	200.76 ± 45.66	1558.87 ± 400.34	100.78 ± 45.62	240.66 ± 98.24	100.42 ± 65.43	60.35 ± 10.92
Propanoic acid	110.45 ± 22.38	750.92 ± 100.27	150.36 ± 20.99	198.26 ± 35.77	120.11 ± 39.90	ND
Butyric acid	200.65 ± 26.21	890.45 ± 76.27	160.91 ± 10.87	155.21 ± 86.10	145.78 ± 14.28	ND
2,3-butanediol	167.26±15.55	300.99 ± 90.81	100.24 ± 26.87	500.25 ± 155.42	321.85 ± 109.26	65.12 ± 10.78
*cis*-3-hexen-1-ol	ND	ND	ND	ULQ	100.76 ± 80.22	ND
Isoamyl alcohol	ULQ	240.99 ± 56.71	105.50 ± 28.91	300.97 ± 109.28	230.89 ± 56.20	500.98 ± 120.90
1-butanol	ULQ	120.98 ± 17.58	100.11 ± 9.87	110.27 ± 4.78	ULQ	200.90 ± 56.72
1-pentanol	ND	ND	ND	ULQ	ULQ	145.87 ± 34.99
Phenylethyl alcohol	100.10 ± 23.98	200.34 ± 55.40	100.21 ± 45.99	450.92 ± 100.21	300.65 ± 145.12	240.98 ± 48.92
Linalool	ND	ND	ND	ULQ	ULQ	ND
Ethyl-L-Lactate	110.99 ± 9.76	230.97 ± 56.78	105.67 ± 20.98	500.98 ± 200.17	300.97 ± 87.22	ULQ
Phenyl ethyl acetate	ULQ	ULQ	ULQ	140.43 ± 32.88	100.35 ± 4.90	ND
Diethyl succinate	ULQ	150.97 ± 34.55	120.97 ± 16.38	206.76 ± 23.76	130.98 ± 76.12	100.24 ± 6.54
Diethyl malate	ULQ	100.65 ± 54.98	ULQ	100.89 ± 23.19	120.97 ± 32.90	ND
Ethyl caprylate	ULQ	ULQ	ULQ	167.98 ± 34.89	104.51 ± 4.31	ND
Hexyl acetate	ND	ULQ	ULQ	ULQ	109.26 ± 3.78	ND

ULQ = under limit of quantitation; ND = not detected.

## Supplementary Materials

The following are available online at www.mdpi.com/2218-1989/7/3/37/s1, Supplementary Table 1: The origin of samples used in this study.

## Abbreviations

The following abbreviations are used in this manuscript:

GC-MS	Gas chromatography-mass spectrometry
LOD	Limit of detection
LOQ	Limit of quantification
CV	Coefficient of variation
RSD	Residual standard deviation
