# Supplementary material for: Rapid Quantification of Major Volatile Metabolites in Fermented Food and Beverages Using Gas Chromatography-Mass Spectrometry
Source: Metabolites. 2017 Jul 27;7(3):37. doi: 10.3390/metabo7030037 (PMC5618322; doi:10.3390/metabo7030037)
Supplement: Supplementary file 1 [file metabolites-07-00037-s001.pdf]

SupplementaryMaterials: Rapid Quantification of Major Volatile Metabolites in Fermented Food and Beverages Using Gas Chromatography-Mass Spectrometry

Farhana R. Pinu and Silas G. Villas-Boas

Supplementary Table 1: The origin of samples used in this study

Sample	Type/variety	Producer/Importer	City	Country
		**Beers**		
Beer 1	Lager	Lion Breweries	Dunedin	New Zealand
Beer 2	Pale lager	DB Breweries	Timaru	New Zealand
Beer3	Indian pale ale	Epic Brewing Company	Auckland	New Zealand
		**White wines**		
White wine 1	2013 Sauvignon blanc	Brancott Estate	Blenheim	New Zealand
White wine 2	2012 Chardonnay	Villa Maria Estate	Auckland	New Zealand
White wine 3	2012 Pinot Gris	Huntaway Reserve	Gisborne	New Zealand
		**Red wines**		
Red wine 1	2012 Merlot	Sileni Estate Winery	Hastings	New Zealand
Red wine 2	2013 Pinot Noir	Rippon Vineyard and Winery	Wanaka	New Zealand
Red wine 3	2012 Syrah	Misson Estate Winery	Napier	New Zealand
		**Whiskies**		
Whisky 1	Single malt	Glenkinchie Distilleries	East Lothian	Scotland
Whisky 2	Blended malt	Diageo	Kilmarnock	Scotland
Whisky 3	Blended malt	Blair Athol Distillery	Pitlochry	Scotland
		**Balsamic vinegars**		
Vinegar 1	–	MazzettiL'originale	Modena	Italy
Vinegar 2	–	MazzettiL'originale	Modena	Italy
Vinegar 3	–	William Aitken & Co.	Modena	Italy
Vinegar 4	–	Pams	Modena	Italy
Vinegar 5	–	Delamaine Fine Foods	Modena	Italy
Vinegar 6	–	Tastemaker Limited	Modena	Italy
		**Sourdough samples**		
Sourdough 1	–	Waikato University	Auckland	New Zealand
Sourdough 2	–	Waikato University	Auckland	New Zealand
Sourdough 3	–	Farro Fresh	Auckland	New Zealand
